# Prediction of Wear Rate of Glass-Filled PTFE Composites Based on Machine Learning Approaches

**DOI:** 10.3390/polym16182666

**Published:** 2024-09-22

**Authors:** Abhijeet R. Deshpande, Atul P. Kulkarni, Namrata Wasatkar, Vaibhav Gajalkar, Masuk Abdullah

**Affiliations:** 1 Department of Mechanical Engineering, Vishwakarma Institute of Information Technology, Pune 411046, India; atul.kulkarni@viit.ac.in (A.P.K.); vaibhav.22110335@viit.ac.in (V.G.); 2 Department of Computer Engineering, Vishwakarma Institute of Information Technology, Pune 411046, India; namrata.kharate@viit.ac.in; 3 Faculty of Engineering, University of Debrecen, Ótemető strt. 2–4, 4028 Debrecen, Hungary

**Keywords:** machine learning, PTFE composite, wear analysis, Pearson’s correlation

## Abstract

Wear is induced when two surfaces are in relative motion. The wear phenomenon is mostly data-driven and affected by various parameters such as load, sliding velocity, sliding distance, interface temperature, surface roughness, etc. Hence, it is difficult to predict the wear rate of interacting surfaces from fundamental physics principles. The machine learning (ML) approach has not only made it possible to establish the relation between the operating parameters and wear but also helps in predicting the behavior of the material in polymer tribological applications. In this study, an attempt is made to apply different machine learning algorithms to the experimental data for the prediction of the specific wear rate of glass-filled PTFE (Polytetrafluoroethylene) composite. Orthogonal array L25 is used for experimentation for evaluating the specific wear rate of glass-filled PTFE with variations in the operating parameters such as applied load, sliding velocity, and sliding distance. The experimental data are analysed using ML algorithms such as linear regression (LR), gradient boosting (GB), and random forest (RF). The R^2^ value is obtained as 0.91, 0.97, and 0.94 for LR, GB, and RF, respectively. The R^2^ value of the GB model is the highest among the models, close to 1.0, indicating an almost perfect fit on the experimental data. Pearson’s correlation analysis reveals that load and sliding distance have a considerable impact on specific wear rate as compared to sliding velocity.

## 1. Introduction

Polymer-based composites are materials that consist of a polymer matrix reinforced with filler materials. The polymer matrix is a continuous phase that provides structural integrity and holds the composite together, while the filler materials, such as fibers, particles, or additives, enhance the mechanical, thermal, electrical, or other properties of the composite [1,2]. Polymer-based composites can be categorised into two main types: thermoset matrix composites and thermoplastic matrix composites. The diversity of polymer composites is due to their lightweight, strength, and durability, making them suitable for applications; however, inherent advantages of polymer-based composites include low density, corrosion resistance, and cost-effectiveness, making them an attractive alternative to metal and other general materials. The choice of polymer matrix and filler materials depends on the desired properties of the composite [3,4]. Common types of polymer matrix materials include epoxy, polyester, polyethylene, and polypropylene, while filler materials can include carbon, glass, nanoparticles, and others. Carbon is widely used because of its high strength-to-weight ratio, making it suitable for applications that require high mechanical performance. While glass provides good tensile strength and is often used in structural components, nanoparticles such as graphene or silica improve electrical conductivity [5,6,7]. Polymer-based composites have a wide range of applications across industries such as aerospace, automotive, construction, electronics, sports equipment, and many other fields where lightweight, strong, and durable materials are required [5,6].

Another crucial area of composite research utilising polymer tribology is the investigation of friction, wear, and lubrication. Tribological performance is crucial in applications where moving parts are subjected to mechanical stress, like gears, bearings, and automobile components. The intricate tribological behavior observed in polymer-based composites is influenced by various elements such as filler type, quantity, and size, as well as external conditions including temperature and humidity. Fillers provide composite materials with a longer lifespan in addition to increasing their resistance to wear [8]. Predicting wear behavior, however, is difficult, and conventional modelling approaches frequently fall short of providing an accurate picture of how polymer composites will behave in practical settings. As a result, extensive experimental testing is required, which is more time-consuming [9].

Recent advances in machine learning (ML) have opened new avenues for addressing the challenge of predicting the behavior of polymer-based composites. Machine learning models can be trained to predict important ternary properties such as wear rate and friction coefficient in relation to material characteristics, filler material and operating conditions. This approach allows researchers to practice finding non-linear relationships between variables and makes it possible to understand physical behavior more comprehensively [10].

The use of machine learning in the research of polymer-based composite materials is a breakthrough. This reduces the need for extensive experimental testing, saving time and costs. It accelerates the development of new composites with optimised ternary properties. This data-driven approach represents a new and innovative way to advance the field of mass science and polymer-based composite design [11]. It also provides more accurate material performance predictions and leads to better-engineered products for a variety of industries.

Although machine learning (ML) shows promise in predicting composite qualities, its incorporation into traditional composite design approaches is still in its early stages. There is a research gap in establishing strong, data-driven frameworks that successfully combine experimental data and machine learning concepts to accurately forecast and improve composite qualities. There is a scarcity of comprehensive, high-quality datasets covering a wide range of filler types, matrix materials, and environmental factors. Researchers can focus on creating and curating such datasets to increase the accuracy of machine learning models.

## 2. Material and Methods

### 2.1. Materials

PTFE filled with 20% glass has been considered for wear testing in this investigation. The acceptable volume percentage of glass in PTFE composites ranges from 10% to 30%; however, 20% is a typical and practical choice. A 20% volume percentage typically results in a significant boost in mechanical characteristics while maintaining the composite’s flexibility and machinability. Higher glass content increases wear resistance while increasing friction and reducing the composite’s self-lubricating characteristics. Hence, PTFE filled with 20% glass is selected for the present study. Table 1 provides the material’s composition. The source of the substance is HM Polymers in Pune.

Table 2 provides information on material qualities. The purchased material is machined and cut to a length of 32 mm and a diameter of 10 mm, making it suitable for holding in a pin holder.

### 2.2. Experiment Details

**Figure 1:**
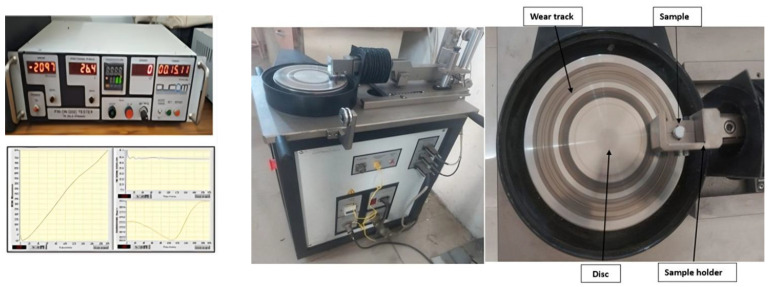
Actual pin-on-disc test setup.

A pin-on-disc setup was used for the dry sliding wear testing. The pin-on-disc test setup procured from Ducom Instruments Pvt. Ltd., Banglore, India, shown in Figure 1, consists of a rotating plate and pin attachment to hold the pin in contact with the plate. The load on the pin was applied through a lever arm. The lever arm is mounted with a load cell and linear variable differential transducer (LVDT) for the measurement of friction and wear, respectively. The specimens have a diameter of 10 mm and a length of 32 mm after being machined with a SimpleTurn 5075 CNC machine Procured from ACE Micromatic, Banglore, India. In the present work, L, SV, and SD are the operating parameters, and their levels are shown in Table 3.

### 2.3. Machine Learning Approach

It is found that the study of friction and wear is empirical and data-driven. The effect of the processing parameters, fillers content, and size on the wear behavior of polymer composite is difficult to evaluate using traditional modelling [10]; however, machine learning (ML) methods can provide a desirable solution to such cases. ML methods make it possible to establish new correlations in tribological data to predict the tribological behavior of materials. This involves structure–property correlations between the composition and structural parameters of composite materials, tribological test conditions, friction, and wear [10,11,12].

**Figure 2:**
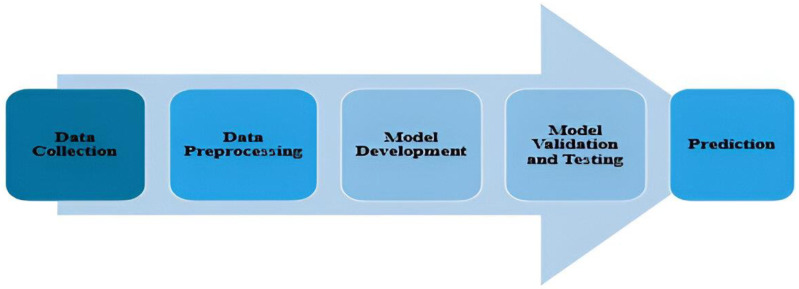
Machine learning workflow.

Figure 2 depicts the machine learning methodology for this work. During the data collecting phase, tribological data are gathered by experiments. The experiments use load (L), sliding velocity (SV), and sliding distance (SD) as independent parameters and wear rate as the dependent parameters. The data preprocessing phase involves data scaling to guarantee that all features contribute equally to the model, which is especially critical for machine learning algorithms that are sensitive to data size. Additionally, data are divided into test and training data in an 80:20 ratio [10].

Various ML approaches, such as LR, RF, and GB, are used in model development. During training, the model learns about the relationships between input features. Models are effectively used to analyse the frictional behavior of polymer composite materials reinforced with glass. During the model validation step, the model’s performance is validated using techniques such as k-fold cross-validation to confirm that it generalises well to new data, and the model’s hyperparameter is tweaked to maximise outcomes. Once validated, the model is used to make predictions using test data.

#### 2.3.1. Linear Regression (LR) Model

The LR model is a supervised machine learning model. Regression is a technique for estimating relationships between the variable and the response when data are generated through different sources [12]. It explains how the variables and responses are correlated with each other. LR is the most basic and popular machine learning algorithm commonly used for predictive analysis. If the predictive response is single, linear regression is preferably used with a linear predictor function to predict the response. However, a multiple regression model is preferred for multiple predictor responses. Linear models are the most basic parametric approaches and should always be given priority because they can address a wide range of problems, including those that are essentially non-linear. A regression is a continuous prediction with a variety of applications; therefore, it is critical to understand how a linear model can fit the data, what its strengths and weaknesses are, and when it is better to choose another option.

In the current study, a preprocessing pipeline was used to prepare data for modelling, which included standardisation using StandardScaler to normalizse the features, polynomial feature generation via Polynomial Features to capture non-linear relationships, and feature selection with SelectKBest using the f_regression scoring function to retain the top features. GridSearchCV was used for hyperparameter tweaking to determine the best degree of polynomial features and the number of top features to choose from. The hyperparameter grid includes polynomial degrees ranging from 1 to 5 and feature selection k values ranging from 5 to 30 [13].

#### 2.3.2. Random Forest

It is a supervised machine learning model that works on small to medium datasets. It is an ensemble of decision trees that is used for prediction and classification. It is an effective technique that employs the prediction skills of numerous independent decision trees. RF, as the name implies, creates a prediction model by collecting distinct decision trees that work on a small set of data [14,15,16,17]. RF uses begging and bootstrapping techniques to overcome the overfitting problem of DTs. RF considers randomisation in the DT building process, preserving the DTs’ uniqueness. The model’s complexity relies on the number of DTs and characteristics employed. RF shows satisfactory performance without proper parameter optimisations.

In this work, the model’s performance is optimised by using GridSearchCV with 5-fold cross-validation. The settings were tuned to include the number of trees (n_estimators), maximum tree depth (max_depth), minimum samples required to split a node (min_samples_split), and minimum samples required at a leaf node. The best-performing model was determined with the following parameters: n_estimators = 100, max_depth = 50, min_samples_split = 2, and min_samples_leaf = 1. This model was chosen because it had the lowest mean squared error (MSE) during the cross-validation phase [13].

#### 2.3.3. Gradient Boosting

It is a supervised machine learning technique that solves classification and regression problems. A set of machine learning algorithms combines numerous weak learning models to create a powerful predictive model. GB is gaining popularity because of its ability to classify difficult datasets [13,14,15,16]. It is an ensemble learning method that trains the model in steps, with each new model seeking to correct the previous model. It combines numerous weak learners to create powerful ones. Each new model is trained using gradient descent to minimise the preceding model’s loss function, which could mean squared error or cross entropy. In each iteration, the technique computes the gradient of the loss function in relation to the current ensemble’s predictions and then trains a new weak model to minimise that gradient [18,19,20]. The new model’s predictions are then added to the ensemble, and the cycle is repeated until the terminating condition is achieved.

To fine-tune the model’s hyperparameters, a grid search was performed using GridSearchCV and 5-fold cross-validation. The parameters investigated included the learning rate, the number of boosting stages (n_estimators), the maximum depth of trees (max_depth), the minimum samples required to split a node (min_samples_split), the minimum samples required at a leaf node (min_samples_leaf), and the number of features considered for splitting (max_features). The grid search yielded the ideal hyperparameters as follows: learning_rate = 0.3, max_depth = 2, max_features = sqrt, min_samples_leaf = 3, min_samples_split = 2, and n_estimators = 200 [13].

#### 2.3.4. Pearson’s Correlation

Pearson’s correlation coefficient, commonly denoted as r, is a statistical measure that quantifies the strength and direction of a linear relationship between two continuous variables. Pearson’s coefficient between 0 and 1 indicates a positive correlation, i.e., when one variable changes, the other variable changes in the same direction. Pearson’s coefficient is 0; it indicates no correlation, i.e., there is no relationship between the variables. Pearson’s coefficient between 0 and −1 indicates a negative correlation, i.e., when one variable changes, the other variable changes in the opposite direction. The closer the coefficient is to ±1, the stronger the linear relationship between the two variables. Despite its widespread use, Pearson’s correlation is a powerful and simple tool for measuring the linear association between two continuous variables. While it provides valuable insights, researchers should be cautious about its assumptions and limitations, particularly regarding causality and sensitivity to outliers.

The Pearson’s correlation coefficient is calculated using the following formula:

(1) $$r=\frac{\sum \left(X_{i}-\bar{X}\right)\left(Y_{i}-\bar{Y}\right)}{\sqrt{\sum {\left(X_{i}-\bar{X}\right)}^{2}\sum {\left(Y_{i}-\bar{Y}\right)}^{2}}}$$

where $X_{i}\;\mathrm{and}\;Y_{i}$ represent individual data points of the variables *X* and *Y,* $\bar{X}\;\mathrm{and}\;\bar{Y}$ are the means of *X* and *Y*, respectively.

## 3. Result and Discussions

Design of Experiment (DOE) is a strong statistical tool for determining the effect of many input parameters on the expected outcome. It is a cost-effective and efficient experimentation technique that minimises the number of trials required while ensuring data integrity. The experiment design in this work is based on an L25 orthogonal array. The tests use three input parameters, L, SV, and SD, each of which has five levels. The experiments were conducted using the L25 orthogonal matrix and repeated twice to guarantee that the results were consistent and accurate. The detailed experiment matrix is shown in Table 4.

**Figure 3:**
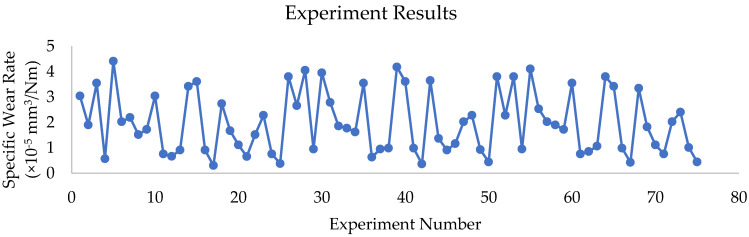
Experiment value of SWR.

Initially, the whole specimen surface was cleaned using acetone and the weight of the pin was measured before and after the experiment for estimating the mass loss. Disc revolutions are set in the control unit as per the required sliding velocity and sliding distance; however, the track diameter was maintained at 130 mm. All the tests were performed under dry sliding conditions. The experiment results are represented in Figure 3.

Experiment results show the minimum wear rate of 3.04186 × 10^−5^ mm^3^/Nm with the L of 150 N, SV 2 m/s, and SD of 5000 m, and the maximum wear rate is observed as 4.410698 × 10^−5^ mm^3^/Nm with the L of 60 N, SV 5 m/s and SD of 5000 m. Experimental data show that, at a lower value of L (60 N), with lower SD (1000 m and 2000 m), SWR fluctuates between 0.570349 × 10^−5^ and 4.410698 × 10^−5^ mm^3^/Nm. This indicates that SWR values are relatively high, indicating higher wear at these conditions. However, as L increases 90 N and above with higher sliding velocity, the formation of stable transfer film makes the surface more resistant to wear [6,7]. It is also observed that, at lower SD (1000 m and 2000 m), rough surface asperities make the material subjected to higher wear. The SWR at 1000 m is 3.802326 × 10^−5^ mm^3^/Nm with lower L (60 N) and SV (1 m/s), which is relatively high. It is evident that at L = 180 N, SV = 5 m/s, and SD = 4000 m, the SWR further decreases to 0.443605 × 10^−5^ mm^3^/Nm. The higher SD led to steady-state wear, where the surface became smooth, which resulted in reducing friction and material removal [1,4]. Due to higher frictional forces and rough counterparts, a higher SWR of 3.802326 × 10^−5^ mm^3^/Nm is observed at lower SV (L = 60 N, SD = 1000 m, SV = 1 m/s). However, a higher SV with a higher L and SD wear rate decreases, which is due to the formation of stable transfer film [11]. Due to surface hardening or more stable contact at greater loads, the experimental evidence points to a pattern where SWR reduces with increasing L and SD. However, because of the greater material removal at lower L and SV, the SWR tends to rise. The specific wear rate is calculated by the equation given below:

(2) $$Specific\;Wear\;Rate=\frac{Volume\;Loss\;\left({\mathrm{mm}}^{3}\right)}{Load\;\left(N\right)\times Sliding\;Distance\;\left(m\right)}$$

In the above equation, volume loss is calculated using the mass loss and density of the material. Mass loss of the material is obtained by measuring the weight of the pins before and after the test.

The present study is to investigate the influence of operating parameters such as load, sliding velocity, and sliding distance on the tribological performance of PTFE (Polytetrafluoroethylene) composites. LR, RF, and GB models are used for the prediction of the specific wear rate of PTFE composite under different working conditions using the data obtained from the experiments. The experiment data are divided into an 80:20 split as training and testing sets, respectively.

**Figure 4:**
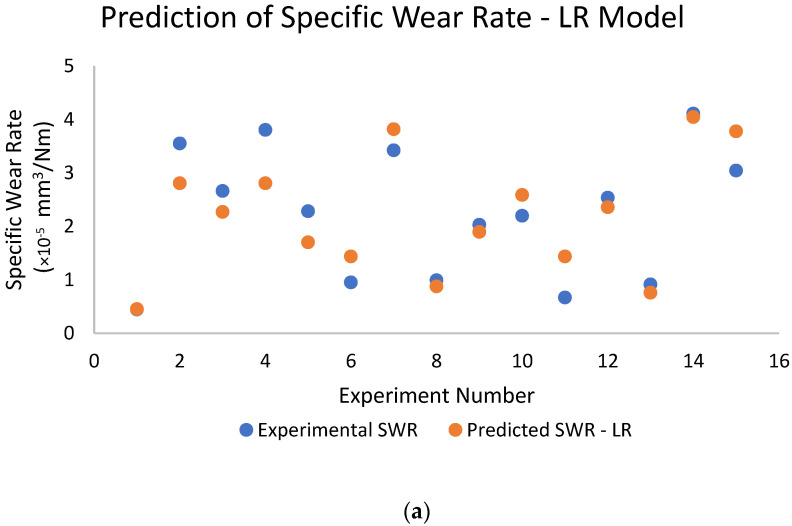

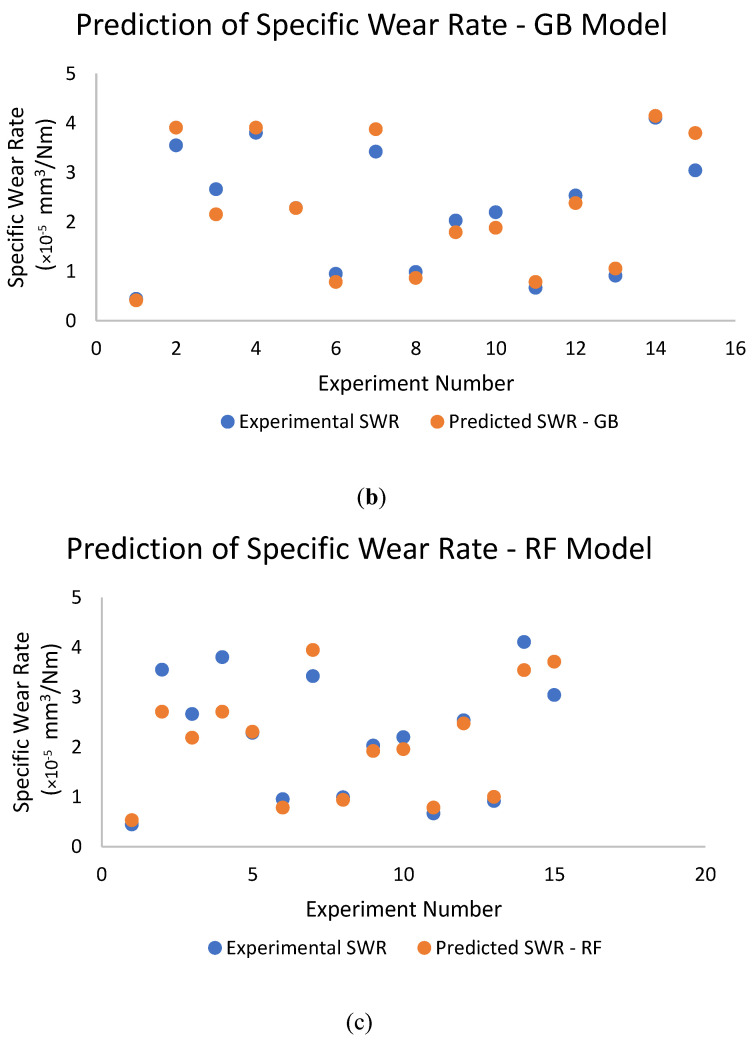
Comparison of experiment and predicted values of SWR. (**a**) LR Model, (**b**) GB Model, (**c**) RF Model.

Figure 4a–c represents the comparison between experimental and predicted values for different models used. Figure 4a shows the experimental and predicted SWR values for the test dataset using the LR model. The test experiments show SWR variation between 0.5 × 10^−5^ and 4.5 × 10^−5^ mm^3^/Nm, whereas the model predicts SWR as between 0.7 × 10^−5^ and 4.2 × 10^−5^ mm^3^/Nm. Figure 4b shows SWR variation using the GB model; experimental SWR is observed between 0.5 × 10^−5^ and 4.5 × 10^−5^ mm^3^/Nm, whereas the model predicts SWR as between 0.45 × 10^−5^ and 4.4 × 10^−5^ mm^3^/Nm. Similarly, Figure 4c shows experimental SWR between 0.5 × 10^−5^ and 4.5 × 10^−5^ mm^3^/Nm, whereas the RF model predicts variation in SWR between 0.4 × 10^−5^ and 4.3 × 10^−5^ mm^3^/Nm. The lack of any discernible trend in the LR model indicates that the model either consistently overestimates or underestimates the SWR, implying that the errors in prediction are distributed randomly. This randomness might indicate noise in the data or limits in the model’s ability to generalise to all test scenarios. Although the LR model approximates the SWR quite well, several discrepancies indicate the need for future improvements, such as the inclusion of non-linear effects [12]. The GB model demonstrates high predictive skills in these settings, as evidenced by its close consistency with the experimental values. In most test experiments, it displays lesser variances and more consistent forecasts. The RF model’s outstanding prediction capabilities are demonstrated by the close agreement between the experimental and predicted SWR [14]. On the other hand, a few trials show slight deviations between the experimental and predicted results in the mid-range SWR values, when the predicted values are nearly like the experimental results. Non-linear relationships are better captured by GB than by LR and RF [16,17,18,19,20]. Its ability to handle complicated datasets and demonstrate greater robustness across tests is its main strength. While GB still exhibits some prediction errors in extreme instances, it performs well with more complex data and relationships.

**Figure 5:**
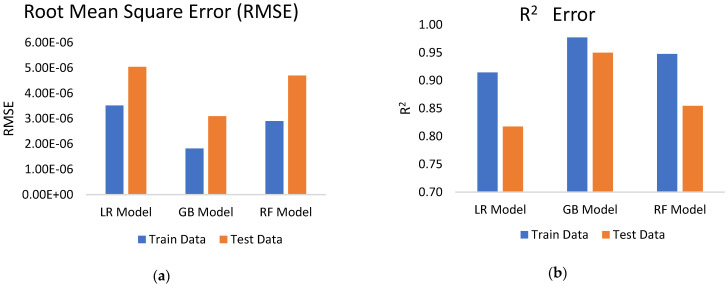
Comparison of (**a**) RMSE, (**b**) R^2^ for different models.

The predictive ability of the models is further investigated using R^2^ and RMSE. R^2^ and RMSE were determined for both training and test data, as shown in Figure 5. RMSE for training data for LR, GB, and RF was observed as 3.52 × 10^6^, 1.82 × 10^−6^, and 2.9 × 10^−6^ respectively. However, test data RMSE was observed as 5.04 × 10^−6^, 3.09 × 10^−6^, and 4.7 × 10^−6^, respectively, for LR, GB, and RF models. The results show that the GB model fits the data effectively, and eventually precisely predicts the SWR. The R^2^ for LR, GB, and RF training data was 0.91, 0.97 and 0.94, respectively. Similarly, the corresponding values of R^2^ for test data were 0.81, 0.94, and 0.85, respectively. The R^2^ value of the GB model for the training data is the highest among the models, close to 1.0, indicating an almost perfect fit on the training set.

The relationship between variables used in the study was investigated using Pearson’s correlation analysis [4,20,21,22]. It is a statistical tool that indicates the strength between the different variables or groups of variables. It also helps in identifying the non-linear relationship between the variables for better predictive analysis. Pearson’s correlation coefficient ranges from −1 to 1, indicating the strength and direction of the relationship between the variables.

**Figure 6:**
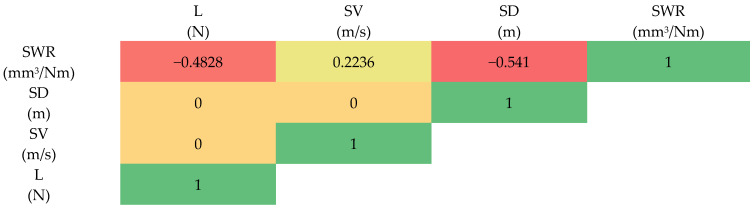
Correlation coefficient.

The correlation coefficient between load, sliding velocity, and sliding distance with specific wear rate is given as −0.4828, 0.2236, and −0.541, respectively, as represented in Figure 6. It indicates that sliding distance has a strong negative correlation with the SWR, whereas load has a moderate negative correlation. However, the sliding velocity shows a weak positive correlation with SWR. It indicates that sliding distance has a greater influence on SWR, followed by load and sliding distance.

## 4. Conclusions

The performance of a glass-filled PTFE composite was experimentally evaluated using a pin-on-disc wear testing machine. The experimental data were analysed using different machine learning algorithms to predict the wear rate of the material under different working conditions. The following conclusions are derived from this study:

- The experimental results demonstrate a minimal wear rate of 3.04186 × 10^−5^ mm^3^/Nm with L of 150 N, SV of 2 m/s, and SD of 5000 m. The highest recorded wear rate was 4.410698 × 10^−5^ mm^3^/Nm, with an L of 60 N, SV of 5 m/s, and SD of 5000 m. The experimental data conclude that wear resistance is improved at higher L and larger SD, as it helps create a stable transfer film, resulting in a lower specific wear rate. However, wear is more erratic at lower L and higher SV as it increases frictional heat, which breaks down the transfer film and is subject to sudden increases in wear rate.
- Machine learning models are effective at predicting wear rate. The gradient boosting algorithm outperforms the random forest and linear regression model. The R^2^ value of the gradient boosting model is 0.97, which is close to 1, and this indicates the perfect fit on the experimental data. Similarly, among all ML models, the lowest RMSE value of 1.82 × 10^−6^ is observed for gradient boosting. This clearly shows that the gradient boosting model may be used to accurately forecast the wear rate of PTFE composites.
- The Pearson’s correlation value of L, SV, and SD with wear rate is observed as −0.4828, 0.2236, and −0.541, respectively. Hence, it is observed that L and SD have a moderate negative impact on wear rate, i.e., wear rate decreases with an increase in L and SD. However, the SV has a weak positive correlation with the wear rate.

## Figures and Tables

**Table 1:** Composition of PTFE composite.

Composition	Volume Fraction (%)	
	PTFE	Glass
1	80	20

**Table 2:** Properties of glass-filled PTFE.

Property	Standard	Value
Colour	Visual	White
Density (g/cc)	ASTM D-638 [7]	2.1–2.3
Tensile Strength (MPa)	ASTM D-638 [7]	17
Hardness (Shore D)	ASTM D-2240 [7]	56–57

**Table 3:** Operating parameters.

Test Parameter Levels			
Level	L (N)	SV (m/s)	SD (m)
1	60	1	1000
2	90	2	2000
3	120	3	3000
4	150	4	4000
5	180	5	5000

**Table 4:** Experiment matrix.

Experiment No	L (N)	SV (m/s)	SD (m)	SWR (×10^−5^ mm^3^/Nm)		
				Trail 1	Trail 2	Trail 3
1	60	1	1000	3.041860	3.802326	3.802326
2	60	2	2000	1.901163	2.661628	2.281395
3	60	3	3000	3.548837	4.055814	3.802326
4	60	4	4000	0.570349	0.950581	0.950581
5	60	5	5000	4.410698	3.954419	4.106512
6	90	1	2000	2.027907	2.788372	2.534884
7	90	2	3000	2.196899	1.858915	2.027907
8	90	3	4000	1.520930	1.774419	1.901163
9	90	4	5000	1.723721	1.622326	1.723721
10	90	5	1000	3.041860	3.548837	3.548837
11	120	1	3000	0.760465	0.633721	0.760465
12	120	2	4000	0.665407	0.950581	0.855523
13	120	3	5000	0.912558	0.988605	1.064651
14	120	4	1000	3.422093	4.182558	3.802326
15	120	5	2000	3.612209	3.612209	3.422093
16	150	1	4000	0.912558	0.988605	0.988605
17	150	2	5000	0.304186	0.365023	0.425860
18	150	3	1000	2.737674	3.650233	3.346047
19	150	4	2000	1.673023	1.368837	1.825116
20	150	5	3000	1.115349	0.912558	1.115349
21	180	1	5000	0.659070	1.166047	0.760465
22	180	2	1000	1.520930	2.027907	2.027907
23	180	3	2000	2.281395	2.281395	2.408140
24	180	4	3000	0.760465	0.929457	1.013953
25	180	5	4000	0.380233	0.443605	0.443605

## Data Availability

Data are contained within the article.
